# Three‐Dimensional Printed Prosthesis Reconstructs Bilateral Type III Pelvic Defect After Malignant Tumors Resection

**DOI:** 10.1111/os.14264

**Published:** 2024-11-24

**Authors:** Xuanhong He, Yi Luo, Chang Zou, Minxun Lu, Yuqi Zhang, Zhuangzhuang Li, Guy Romeo Kenmegne, Yong Zhou, Li Min, Chongqi Tu

**Affiliations:** ^1^ Department of Orthopedics Orthopedic Research Institute, West China Hospital, Sichuan University Chengdu China; ^2^ Department of Model Worker and Innovative Craftsman West China Hospital, Sichuan University Chengdu Sichuan China

**Keywords:** bilateral pubic bone defect, prosthesis, pubic malignant tumors, reconstruction, three‐dimensional printed

## Abstract

**Objective:**

Type III hemipelvectomy and reconstruction are challenging. Several reconstruction options, including autologous soft tissue, prosthesis patch, autologous, or allograft, were reported, but a variety of shortcomings limited their application. Three‐dimensional (3D)‐printed prosthesis was designed to reconstruct the unilateral Type III pelvic defect and had favorable clinical outcomes. However, the reconstruction method for bilateral Type III pelvic defect was few reported. This study aims to design a bilateral pubis prosthesis and evaluate the early clinical outcomes and complications.

**Methods:**

We retrospectively collected patients receiving 3D‐printed bilateral pubis prosthesis reconstruction after malignant tumor resection between 2017 and 2021. Demographics, anatomic data, operation time, blood loss, and clinical outcomes of patients were analyzed. The Musculoskeletal Tumor Society‐93 (MSTS‐93) score was performed to evaluate the function and complications were recorded.

**Results:**

Four patients, including three for females and one for males, were enrolled in this study. Prosthesis was designed according to the pelvic anatomical data. The mean operation time and blood loss were 308.8 min (range, 240–400 min) and 655 mL (range, 400–1100 mL), respectively. The average follow‐up was 15.5 months (range, 12–16 months). The mean MSTS was 28.5 (28–29). One patient had incision necrosis postoperatively. No hernia, prosthesis displacement, or implant failure occurred during follow‐up. Four patients with 15 interfaces showed good osteointegration.

**Conclusions:**

3D‐printed bilateral pubis prosthesis could restore the integrity and stability of pelvic ring and improve limb function. Meanwhile, this reconstruction option provided a rigid bony‐soft support to prevent the development of hernia. In all, 3D‐printed bilateral pubis prosthesis is recommended to be a favorable selection for Type III pelvic defect reconstruction.

## Graphical abstract

In this study, personalized bilateral pubis prosthesis was designed and printed by 3D printing technology to reconstruct the pelvic ring and prevent hernia. During the follow‐up, patients had considerable early clinical outcomes and no hernia.

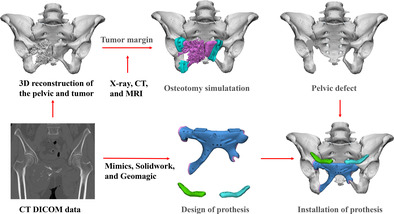

## 1 Introduction

As described by Enneking and Dunham, Type III resection involves the partial or complete resection of the pubis [1]. In more cases, the Type III bone defect would not be reconstructed because the weight‐bearing axis is maintained [2]. However, the defect of anterior pelvic ring may lead to the increase of shear force and vertical tension of sacroiliac joint, which may lead to joint overactivity and osteoarthritis [3, 4]. Besides, the removal of malignant tumors involving Type III pelvis always causes the extensive bone and sizable soft tissue defects, resulting in hernia [5]. Therefore, reconstruction after Type III pelvic resection of tumor seems necessary.

Several reconstruction methods after Type III pelvic resection were reported. Autologous tissues such as myocutaneous flap, aponeurosis, and ligament can be applied for pelvic reconstruction because of the availability and biocompatibility [6, 7, 8]. However, the additional injury and insufficient soft tissue after extensive tumor resection are problems that need to be faced. In addition, artificial material such as prosthetic mesh is also used to reconstruct the pelvic ring to prevent the formation of hernia [5, 9]. However, potential infection or rejection of the artificial patch could be severe complications. And, those non‐bony materials may not provide enough rigidity or durability to prevent herniation [10]. More importantly, non‐bony materials reconstruction may lead to changes in pelvic structure and mechanics, resulting in acetabular displacement, sacroiliitis, and other complications [3, 4]. In some studies, autografts and allografts are used in the repair of pelvic defects to achieve good bone reconstruction [11]. However, complications such as graft infection, failure, and displacement restrict its application [11, 12, 13].

As a result of the development of three‐dimensional (3D)‐printed technology, bone defects caused by tumors are gradually repaired with personalized implants [14]. 3D‐printed prosthesis can not only anatomically reconstruct the bone defect and restore the integrity of pelvic ring, but also provide the anchorage point for soft tissue suture to prevent hernia [13, 15]. At present, there are few reports on the application of 3D‐printed prostheses in the reconstruction of pure Type III pelvic defects. Our center has previously reported a novel 3D‐printed prosthesis for the reconstruction of unilateral pubic defects, which has achieved favorable early outcomes [15, 16]. However, the reconstruction method of bilateral pubic defects has not been reported yet. This study aims to: (i) introduce a novel reconstruction strategy for bilateral Type III bone defect with 3D‐printed bilateral pubic prosthesis and (ii) evaluate the early clinical outcome and related complications.

## 2 Patients and Methods

### 2.1 Patients

**FIGURE 1:**
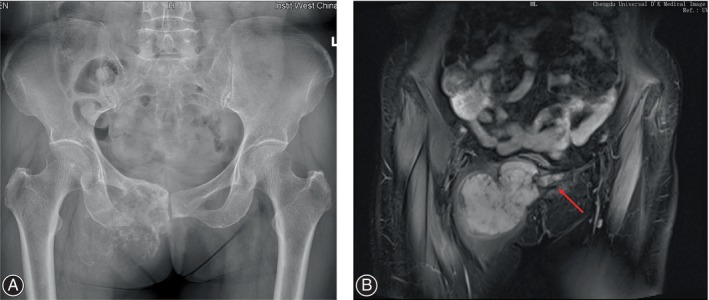
The X‐ray (A) and the MRI (B) demonstrated that the tumor invaded bilateral pubic (red arrow).

We retrospectively analyzed the patients who underwent bilateral Type III malignant tumor resection and 3D‐printed prosthesis reconstruction in our institution from January 2017 to January 2021. Four patients were included in this study with three females and one male. The average age of patients undergoing surgery was 42 years (32–66 years). All patients received X‐rays, computerized tomography (CT), magnetic resonance imaging (MRI), and single‐photon emission computed tomography (SPECT) to evaluate the tumor margin (Figure 1). Fine needle or open biopsy was performed to determine the nature of the tumors. In this study, three patients were diagnosed with chondrosarcoma and one was diagnosed with osteosarcoma. No lung metastasis or bone metastasis were detected (Table 1).

**TABLE 1:** Demographics and clinical outcomes of four patients.

Patients	Sex	Histology	Resection	OT (min)	BL (mL)	MSTS‐93	Oncologic outcome	Complications	Follow‐up (months)
1	F	Chondrosarcoma	Bilateral Type III	240	400	29	NED	—	16
2	M	Chondrosarcoma	Bilateral Type III	325	500	28	NED	—	14
3	F	Osteosarcoma	Bilateral Type III	400	1100	28	NED	Incision necrosis	20
4	F	Chondrosarcoma	Bilateral Type III	270	620	29	NED	—	12
Mean	—	—	—	308.8	655	28.5	—	—	15.5

Abbreviations: BL, blood loss; NED, no evidence of disease; OT, operation time.

The studies were reviewed and approved by the Ethics Committee of West China Hospital, Sichuan University. Written informed consent to participate in this study was provided by the participants.

### 2.2 Anatomic Data Measurement

The 3D models of tumor and pelvis were reconstructed and the anatomic data, including diameter of narrowest part and length of superior pubis and inferior pubis, and the height of pubic symphysis, were measured. The resection margin was identified according to the X‐ray, CT, MRI, and SPECT.

### 2.3 Prosthesis Design and Fabrication

**FIGURE 2:**
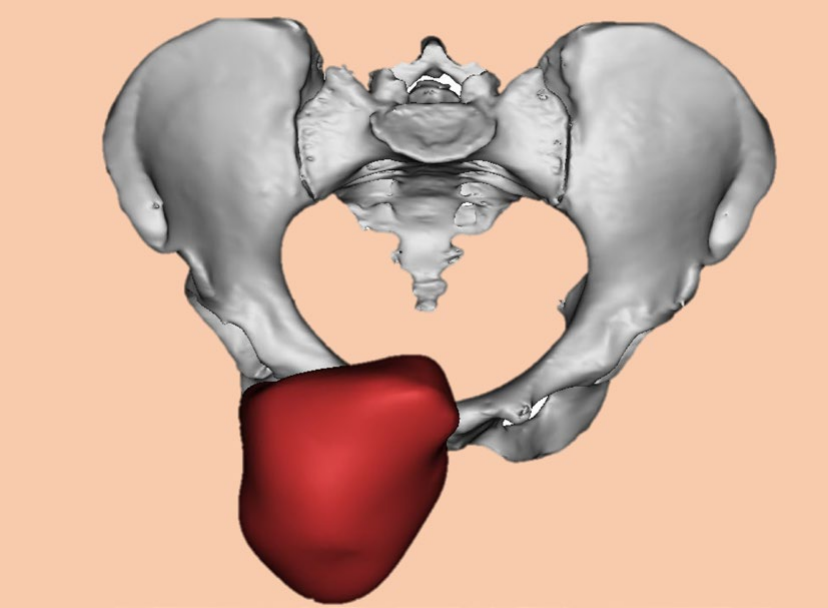
The 3D model of the pelvic and tumor was reconstructed and the tumor involved bilateral pubis.

**FIGURE 3:**
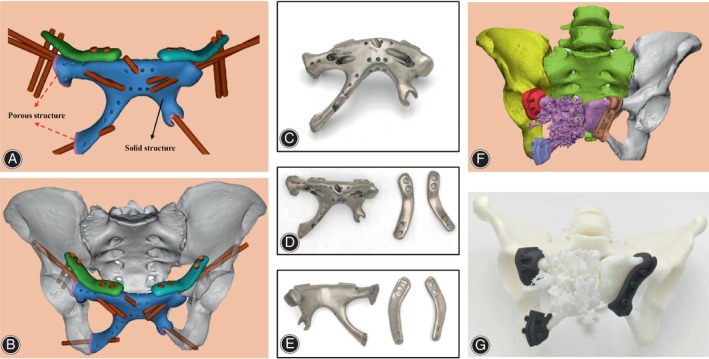
The design (A and B) and the fabrication (C–E) of the 3D‐printed bilateral pubis prosthesis. The prosthesis consists of solid (black arrow) and porous structures (red arrow). Two plates were designed to provide auxiliary initial stability if necessary. Screw holes on the prosthesis are intended for prosthesis fixation, while preset suture holes facilitate muscle and patches suture. The guide plate was designed (F) and fabricated (G) according to the resection range.

Our clinical team designed all prostheses and Chunli Co., Ltd., Tongzhou, Beijing, China fabricated them. The specific design and fabrication process of prosthesis was similar to our previous research [15, 16]. According to the CT and MRI data, we constructed the 3D model of the tumor with Mimics software (Materialize Corp., Leuven, Belgium) (Figure 2). After determining the osteotomy plane according to the tumor margin, we designed the osteotomy guide plate and prosthesis. The fixation grooves of steel plates were present at both sides of the prosthesis, and whether to add steel plates to increase the temporary stability of the prosthesis depending on the intraoperative situation. Suture holes were added to the prosthesis to provide anchorage points for patches or muscles. The contact surface between the prosthesis and the remained bone was designed as a porous structure with a pore size of 600 μm and a porosity of 70% (Figure 3A,B).

All the prostheses were fabricated by electron beam melting technology (ARCAM Q10plus, Mölndal, Sweden). The osteotomy guide plates and the plastic trial model were fabricated by the tereolithography appearance technique (UnionTech Lite 450HD, Shanghai, China) (Figure 3C–E). The prosthesis is fabricated using a 3D printing material of titanium alloy (Ti6Al4V), while the guide plate and the trial model are constructed from nylon.

### 2.4 Surgical Techniques

**FIGURE 4:**
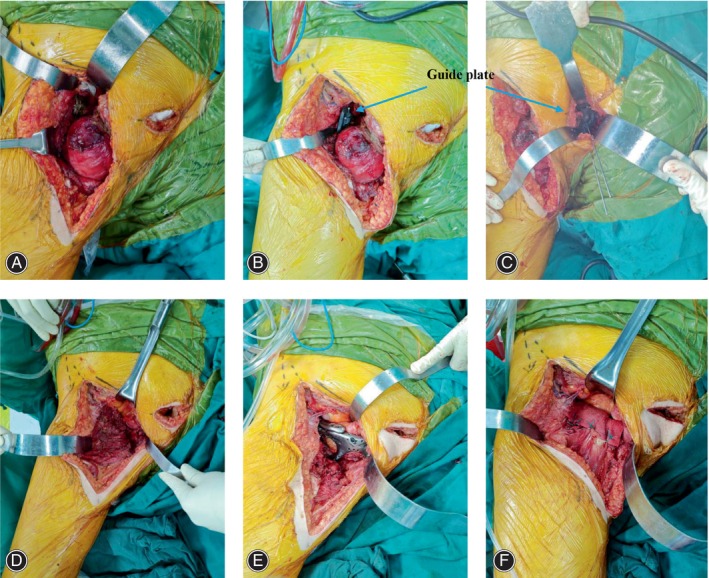
The bilateral pubis tumor was exposed (A) and the guide plates were installed in bilateral pelvis to guide osteotomy (B and C, blue arrow). After resection of the tumor (D), the prosthesis was installed (E), and the patches and muscles were sutured to the prosthesis (F).

In four patients, ilioinguinal incision and transverse suprapubic incision were made to sufficiently expose the tumors. Based on the preoperative imaging examinations, the tumor was completely removed under the guidance of the osteotomy guide plates. Then, a plastic test model with the same size as the prosthesis was used to test whether the prosthesis matched the bone defect. After that, the 3D‐printed prosthesis was installed and precisely fitted to the bone defect. Worth noticing, the screws of the superior pubic branch should avoid penetrating into the hip joint and causing arthritis. After repeated pulsatile lavage with saline and soaking in 10% povidone‐iodine, the patches and muscles were sutured to the suture point of the prosthesis to prevent the hernia of pelvic tissues and organs (Figure 4). Operation time and blood loss during operation were recorded.

### 2.5 Postoperative Management

Postoperative management of the patients with bilateral pubic prosthesis reconstruction was similar to that of the patients undergoing unilateral pubic prosthesis without stem reconstruction we reported before [15, 16]. Regular X‐ray and tomosynthesis‐shimadzu metal artifact reduction technology (T‐SMART) were recommended to all the patients to assess the prosthesis position and the osseointegration between the prosthesis and the host bone. Besides, the Musculoskeletal Tumor Society‐93 (MSTS‐93) score was performed to evaluate the function outcome. Complications, including hernia, incision necrosis, infection, implant failure, or displacement, were recorded.

## 3 Results

### 3.1 Patients

All the patients experienced tumor resection and pelvic ring reconstruction with 3D‐printed bilateral pubic prosthesis. Among four patients, the mean operation time and blood loss were 308.8 min (range, 240–400 min) and 655 mL (range, 400–1100 mL), respectively. The average follow‐up was 15.5 months (range, 12–16 months) (Table 1).

### 3.2 Anatomic Data and Prosthesis Information

The design of the 3D‐printing prosthesis followed the anatomical data of the patients. For the superior pubis, the mean diameter of narrowest part was 10.69 mm (range, 9.89–11.48 mm) and 11.20 mm (range, 10.97–11.42 mm) at the left side and the right side, respectively. The mean length of the superior pubis was 64.24 mm (range, 61.10–66.22 mm) and 62.33 mm (range, 60.41–64.25 mm) at the left side and the right side, respectively. For the inferior pubis, the mean diameter of narrowest part was 7.40 mm (range, 7.33–7.45 mm) and 7.49 mm (range, 7.27–7.63 mm) at the left side and the right side, respectively. The mean length of the inferior pubis was 86.75 mm (range, 84.94–88.62 mm) and 85.64 mm (range, 84.11–87.89 mm), respectively. The mean height of the pubic symphysis was 34.81 mm (Table 2).

**TABLE 2:** The anatomic data of four patients.

Patients	Superior pubis/mm				Inferior pubis/mm				Height of pubic symphysis/mm
	Diameter of narrowest part		Length		Diameter of narrowest part		Length		
	L	R	L	R	L	R	L	R	
1	10.26	10.97	64.26	64.25	7.41	7.27	84.94	84.11	34.14
2	11.48	—	65.36	—	7.45	—	86.69	—	36.27
3	11.12	11.42	61.10	60.41	—	7.58	—	84.93	35.61
4	9.89	—	66.22	—	7.33	7.63	88.62	87.89	33.21
Mean	10.69	11.20	64.24	62.33	7.40	7.49	86.75	85.64	34.81

*Note*: “L” means left; “R” means Right; “—” means unmeasurable diameter because of the tumor bone defect.

### 3.3 Function, Complications, and Imaging Manifestations

At last follow‐up, all the patients could ambulate without assistive devices. The mean MSTS‐93 score was 28.5 (range, 28–29). There was no hernia or infection during the follow‐up. One patient with osteosarcoma suffered from incision necrosis, but recovered after repeated dressing changes. No local recurrence or distal metastasis was found during the follow‐up (Table 1).

**FIGURE 5:**
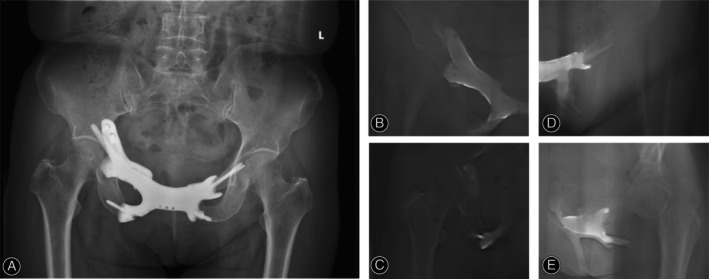
At 6 months of follow‐up, the X‐ray (A) demonstrated no prosthesis displacement or implant failure, and the T‐SMART (B–E) of the prosthesis showed that the osteointegration in the bone–prosthesis interface was good and no significant interfacial gap was observed.

According to the X‐ray at the last follow‐up, no prosthesis displacement or implant failure occurred (Figure 5A). As demonstrated on the T‐SMART, the absence of interfacial gap between bone and implant was observed 6 months postoperatively, implying the good osseointegration (Figure 5B–E).

## 4 Discussion

In this study, we designed a novel 3D‐printed prosthesis to reconstruct the bilateral Type III pelvic defect after malignant tumor resection. Through detailed preoperative planning and prosthesis design achieved with 3D technology, all patients received tumor resection and prosthesis installation. During follow‐up, patients exhibited good functional outcomes with no incidence of prosthesis‐related complications or herniation. Radiographic evaluations indicated proper prosthesis position and good osseointegration at the bone–prosthesis interface. This study initially explored the clinical efficacy, complications, and osseointegration of 3D‐printed prostheses for repairing pelvic type III defects caused by malignant tumors, preliminarily affirming that 3D‐printed prostheses are a feasible strategy for reconstructing pelvic Type III defects after malignant tumors resection.

### 4.1 3D‐Printed Technology in Reconstructing Type III Pelvic Defect

Reconstruction following Type III hemipelvectomy is essential for restoring pelvic ring integrity and stability, as well as for preventing hernia [3, 4, 6, 7]. Some attempts have been made to reconstruct Type III pelvic defects, but challenges persist in concurrently addressing soft tissue reconstruction and osseous structure restoration (Table 3). In recent years, with the development of 3D‐printed technology, 3D‐printed customized prostheses have been applied in the reconstruction of pelvic defects [18]. Zhang et al. designed a 3D‐printed customized prosthesis for unilateral pubis defect reconstruction, which could improve the MSTS score, reduce the VAS score, and prevent the formation of hernia in patients [15, 16]. In this study, we designed a 3D‐printed bilateral pubis prosthesis to reconstruct the bilateral Type III pelvic defect. There are several advantages of 3D‐printed prosthesis in reconstructing Type III pelvic defects. First, the 3D‐printed prosthesis is designed based on the CT data of the patient, which can accurately match the pelvic defect, restore the integrity and stability of the pelvic ring and improve the postoperative function. According to the X‐ray, the implant was well‐fixed (Figure 5A). At the same time, during the follow‐up, four patients receiving pelvic reconstruction had an average MSTS score of 28.5 (Table 1). Second, suture hole added to prosthesis could provide the anchorage point for soft tissue to prevent hernia. For example, adductor longus and brevis, internal oblique, and external oblique muscles were reattached on the prosthesis to avoid the development of hernia [16]. Also, additional patches or artificial ligaments can also be added to provide maximal barrier precaution of hernia (Figure 4F). In this study, no patient found hernia during the follow‐up, implying that 3D‐printed bilateral prosthesis combined with patches or artificial ligaments could be an alternative reconstruction method for bilateral Type III pelvic defect. Thirdly, the prosthesis was composed of solid and porous parts, which could ensure the prosthesis strength and the osteointegration in the interface to provide initial stability and promote bone ingrowth, respectively. As shown, a total of 15 interfaces in our patients demonstrated good osteointegration between the prosthesis and host bone (Figure 5B–E).

**TABLE 3:** Reconstruction methods after Type III pelvic resection.

Author	Type of resection	Reconstruction methods	Number of patients	Follow‐up (months)	Limb function	Complications
Imanishi et al. [8]	Unilateral Type III	Fascia lata transplantation	1	21	MSTS: 29	No
Reddy and Bloom [9]	Unilateral Type III	Marlex mesh	8	114	Enneking classification system: 62% excellent and 37% good result.	1 skin necrosis; 1 leg edema
Du et al. [5]	Unilateral Type III	Pedicled sartorius flap and mesh	12	13.5	MSTS: 29	1 wound dehiscence
Freitas et al. [17]	Type II + III	Autologous fibular graft	2	N/A	N/A	N/A
Karim et al. [13]	Unilateral Type III	Allograft	15	26.8	N/A	2 infections; 1 hernia; 1 hip instability; 1 dislocated total hip arthroplasty; 1 graft failure
Zhang et al. [15, 16]	Unilateral Type III	3D‐printed customized prostheses	5	24	23.6	1 erectile dysfunction
Murphy et al. [10]	Bilateral Type III	Autologous iliac crest graft	1	6	N/A	No
This study	Bilateral Type III	3D‐printed customized prostheses	4	15.5	MSTS: 28.5	1 skin necrosis

### 4.2 Difference Between Unilateral and Bilateral Type III Pelvic Defect

Compared with unilateral Type III pelvic defect, bilateral Type III pelvic defect means massive bone defect and extensive soft tissue loss, which leads to greater difficulty in reconstruction [10, 13]. To the best of our knowledge, there has only been one reported case of resection and reconstruction of bilateral Type III pelvic defect [10]. According to Benjamin et al., autologous iliac bone was sutured to the adjacent pubis with arthrex fiber after bilateral pubis resection [10]. However, the failure and infection of the implant and unnecessary injury in this method are worrying. And, the pelvic ring is still incomplete and may lead to sacroiliitis and dislocation of hip joint. In this study, the 3D‐printed bilateral pubis prosthesis could reconstruct the bone defect and effectively prevent the hernia. Worth noticing in male patients with tumors involving Type III region, erectile dysfunction should be avoided during the tumor resection and reconstruction [15]. In the bilateral pubis prosthesis, the pubic symphysis part of prosthesis was designed with a smaller size than the original bony structure to prevent erectile dysfunction (Figure 3). In addition, different from unilateral pubis prosthesis, we did not reconstruct the movable pubic symphysis out of strength and stability concerns (Figure 3). Nonetheless, no complications such as sacroiliitis, dislocation of hip joint, prosthesis, and screws loosening occurred during the follow‐up in this study. For pelvic defects involving Type III, whether movable symphysis pubis in prosthesis should be designed remains unclear and further exploration and extended follow‐up was needed [18, 19, 20, 21].

### 4.3 Complications

Infection is a significant complication in the surgical treatment of pelvic malignant bone tumors, which is especially true in patients receiving chemotherapy [22]. Therefore, the correction of systemic condition before surgery, strict sterile operation, repeated irrigation with saline and 10% povidone‐iodine solution, and the application of antibiotics are the main measures to prevent the infection. One patient in this study had incision necrosis and healed satisfactorily with repeated dressing changes, which may have been caused by the extensive separation that disrupted blood supply, and the chemotherapy that decreased immunity.

### 4.4 Limitations and Prospect

We have to admit that our study has some shortcomings. Limited by the rarity of malignant tumors invading bilateral pubis, there were fewer cases and shorter follow‐up in our study. At a later stage, we will enroll more patients with bilateral pubis malignant tumors to further validate the clinical efficacy and complications of this prosthesis. Meanwhile, dynamic biomechanical testing before 3D‐printed prosthesis implantation is meaningful, in which the clinicians could optimize implant design according to the biomechanical testing results. In our subsequent research, we plan to conduct biomechanical testing of preoperative implants for each patient to refine the implants, provided that the patient's condition is well‐controlled and the testing does not delay the timing of surgery or affect the chemotherapy regimen. Moreover, for pelvic defects involving Type III, whether movable symphysis pubis in prosthesis should be designed remains unclear. Thus, further exploration of the difference in biomechanics effect and clinical results between rigid and non‐rigid fixation for the pubic symphysis is needed.

## 5 Conclusion

3D‐printed bilateral pubis prosthesis could restore the integrity and stability of pelvic ring and improve limb function. Meanwhile, this reconstruction option provided a rigid bony‐soft support to prevent the development of hernia. In all, 3D‐printed bilateral pubis prosthesis is recommended to be a favorable selection for Type III pelvic defect reconstruction.

## Author Contributions

X.H., L.M., and C.T. designed the research study. L.M. and C.Z. performed the research. Y.Z. provided help and advice on revising the manuscript. Z.L., G.R.K., Y.L., and Y.Z. analyzed the data. X.H. and L.M. wrote the manuscript. All authors contributed to editorial changes in the manuscript. All authors read and approved the final manuscript.

## Disclosure

The authors have nothing to report.

## Ethics Statement

This study was approved by the Ethical Committee of West China Hospital. All the patients agreed to participate in this study and signed the written informed consent.

## Conflicts of Interest

The authors declare no conflicts of interest.

## Supporting information


**Figure S1.** Preoperative tumor 3D models were reconstructed, and the resection margins were determined based on X‐ray, MRI, and SPECT imaging.

**Figure S2.** Postoperative pelvic and implant were reconstructed using 3D CT imaging, consistent with preoperative planning. The implant position was satisfactory with no evident loosening or fractures observed.

**Figure S3.** Preoperative MRI imaging indicates the tumor is primarily located on the right pubic and formed a soft tissue mass, with involvement of the contralateral pubic (red arrows).
