# Supplementary figures and images for: Three‐Dimensional Printed Prosthesis Reconstructs Bilateral Type III Pelvic Defect After Malignant Tumors Resection
Source: Orthop Surg. 2024 Nov 24;17(1):260–8. doi: 10.1111/os.14264 (PMC11735349; doi:10.1111/os.14264)

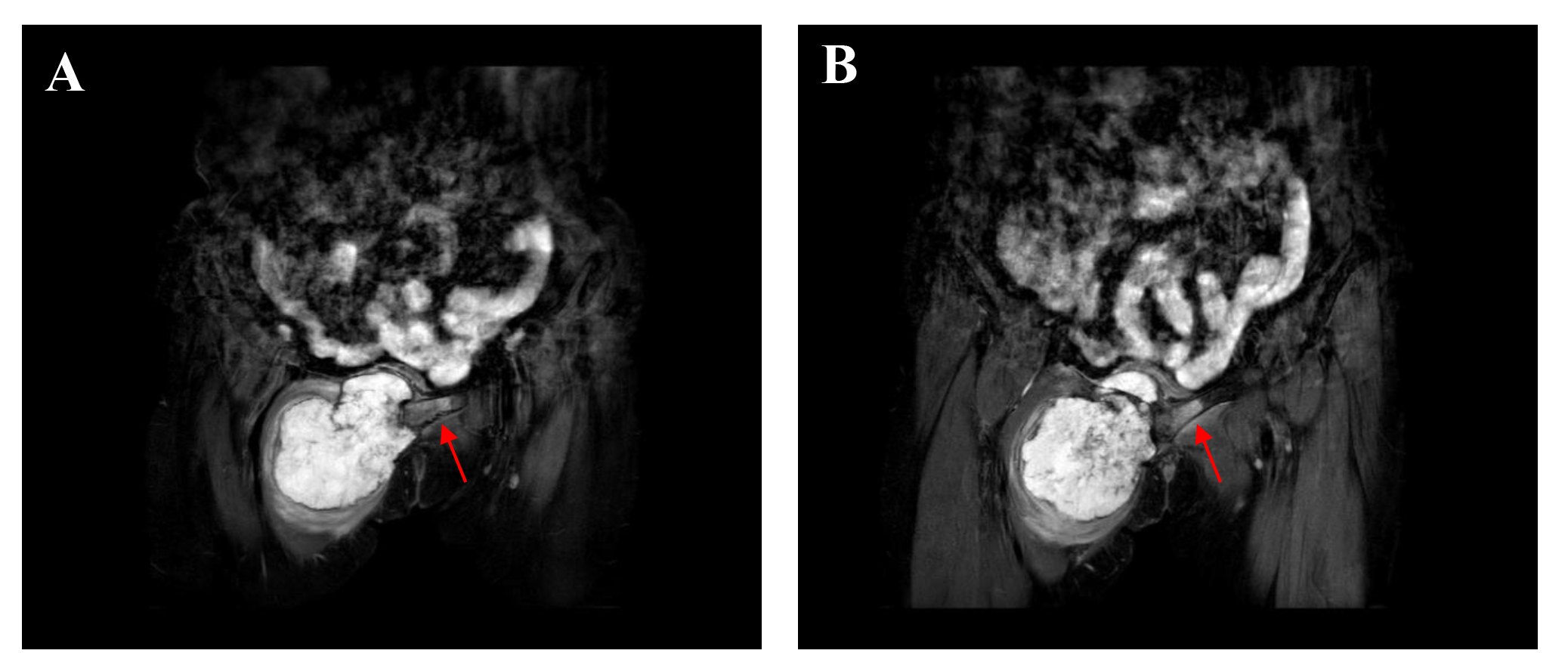

Supplement: Supplementary file 3 — Figure S3. Preoperative MRI imaging indicates the tumor is primarily located on the right pubic and formed a soft tissue mass, with involvement of the contralateral pubic (red arrows). [file OS-17-260-s003.tif]
